# Thyrotoxicosis and dilated cardiomyopathy in developing countries

**DOI:** 10.1186/s12902-021-00796-5

**Published:** 2021-06-28

**Authors:** Bisrat Tesfay Abera, Merhawit Atsbha Abera, Gebretsadik Berhe, Girmatsion Fisseha Abreha, Hirut Teame Gebru, Hiluf Ebuy Abraha, Mohamedawel Mohamedniguss Ebrahim

**Affiliations:** 1 College of Health Sciences, Mekelle University, Mekelle, Ethiopia; 2 College of Health Sciences, Adigrat University, Adigrat, Ethiopia

**Keywords:** β-Blocker, Dilated Cardiomyopathy, Ethiopia, Methimazole, Propylthiouracil, Thyrotoxicosis

## Abstract

**Background:**

Thyrotoxicosis is the state of thyroid hormone excess. But, in sub-Saharan Africa (SSA), specifically Northern Ethiopia, scientific evidence about thyrotoxicosis and its cardiac complications like dilated cardiomyopathy is limited. Therefore, this study aimed to explore the thyrotoxicosis presentation and management and identify factors associated with dilated cardiomyopathy in a tertiary hospital in Northern Ethiopia.

**Methods:**

An institution-based cross-sectional study was conducted in Ayder Comprehensive Specialized Hospital from 2017 to 2018. Data from 200 thyrotoxicosis cases were collected using a structured questionnaire. After describing variables, logistic regression was conducted to identify independent predictors of dilated cardiomyopathy. Statistical significance was declared at *p* < 0.05.

**Results:**

Mean age at presentation of thyrotoxicosis was 45 years and females accounted for 89 % of the cases. The most frequent etiology was multinodular toxic goiter (51.5 %). As well, the most common symptoms and signs were palpitation and goiter respectively. Thyroid storm occurred in 6 % of the cases. Out of 89 patients subjected to echocardiography, 35 (39.3 %) of them had dilated cardiomyopathy. And, the odds of dilated cardiomyopathy were higher in patients who had atrial fibrillation (AOR = 15.95, 95 % CI:5.89–38.16, *p* = 0.001) and tachycardia (AOR = 2.73, 95 % CI:1.04–7.15, *p* = 0.040). All patients took propylthiouracil and 13.0 % of them experienced its side effects. Concerning β-blockers, propranolol was the most commonly (78.5 % of the cases) used drug followed by atenolol (15.0 %). Six patients underwent surgery.

**Conclusions:**

In developing countries like Ethiopia, patients with thyrotoxicosis have no access to methimazole which is the first-line anti-thyroid drug. Besides, they greatly suffer from dilated cardiomyopathy (due to late presentation) and side effects of propylthiouracil. Therefore, we recommend that patients should get adequate health information about thyrotoxicosis and anti-thyroid drugs including their side effects. Additionally, hospitals and other concerned bodies should also avail of TSH tests and methimazole at an affordable cost. Furthermore, community awareness about iodized salt and iodine-rich foods should be enhanced.

## Background

The term thyrotoxicosis refers to the hypermetabolic clinical syndrome resulting from serum elevations in thyroid hormone levels, specifically free thyroxine (T4) and/or triiodothyronine (T3) [1, 2]. The commonly mentioned causes of thyrotoxicosis are Graves’ disease, toxic multinodular goiter, and toxic adenoma. In iodine replete communities, the prevalence of thyrotoxicosis in women is thought to be between 0.5 and 2 %, and 10 times more common than in men [3]. But its prevalence among patients with thyroid disorders may reach up to 62 % [1].

The usual manifestations of thyrotoxicosis are hyperactivity, irritability, dysphoria, heat intolerance, sweating, palpitation, fatigue, weight loss with increased appetite, diarrhea, polyuria, oligomenorrhea, and loss of libido [4, 5]. Also, patients with thyrotoxicosis could develop several complications one of which is thyrocardiac disease that can happen in 27 % of the cases [6]. Some of the cardiac complications of thyrotoxicosis are atrial fibrillation, non-specific ST-T changes, ventricular hypertrophy, and dilated cardiomyopathy [6–9]. Furthermore, 1 % of patients with thyrotoxicosis could present with a life-threatening complication called thyroid storm which has a mortality risk of 10–20 % [10, 11].

The thionamide drugs (propylthiouracil (PTU), methimazole, and Carbimazole), beta-blockers, radioactive iodine, and surgery are the treatment modalities used in the management of thyrotoxicosis and thyroid storm each with different success rates. Patients with thyroid storm will need further treatment with glucocorticoids and Lugol’s solution [1, 12–17].

However, in sub-Saharan Africa (SSA), specifically Northern Ethiopia, scientific evidence about thyrotoxicosis and its complications like dilated cardiomyopathy is limited. Therefore, this study aims to explore the pattern, clinical manifestation, complication, and management of patients with thyrotoxicosis including factors associated with dilated cardiomyopathy in Ayder Comprehensive Specialized Hospital which is the only tertiary hospital in Tigrai, Northern Ethiopia.

## Methods

### Study area and period

 An institution-based cross-sectional study was conducted in Ayder Comprehensive Specialized Hospital (ACSH) which is located in Mekelle, Tigray regional state, Northern Ethiopia. This hospital provides both referral and non-referral services to a 9 million population in its catchment areas of the Tigray, Afar, and North-eastern parts of the Amhara Regional States including the Eritrean refugees. It offers a broad range of medical services to both inpatients and outpatients of all age groups [18]. One of the outpatient service units in this hospital is an endocrine clinic that started to provide services in January 2017. Services in this clinic are given by an endocrinologist, internal medicine residents, and clinical nurses. This study was carried out from December 2017 to June 30, 2018.

### Source and study population

#### Source population

All patients with thyrotoxicosis who had follow-up at the endocrine clinic at ACSH and received anti-thyroid drugs.

#### Study population

All patients with thyrotoxicosis who had follow-up at the endocrine clinic at ACSH during the study period and are on anti-thyroid drugs.

### Eligibility criteria

Patients above 18 years of age with thyrotoxicosis who had follow-up at the endocrine referral clinic of ACSH were involved in this study. All patients included in this study were treated for thyrotoxicosis.

### Samples

All patients who visited the endocrinology clinic and received antithyroid drugs during the study period were included.

### Variables

#### Dependent variables

The dependent variable is dilated cardiomyopathy.

#### Independent variables

Socio-demographic factors (age, gender, occupation, income, educational status, residence), clinical features (symptoms and signs), comorbidity, level of T4, and frequency of TSH follow-up were considered as independent variables.

### Data collection

Data were collected by two trained internal medicine residents through an interviewer-administered structured questionnaire. The questionnaire was designed to collect data related to sociodemographic characteristics, the clinical presentation of thyrotoxicosis, investigation results like biochemical thyroid hormone assessment and fine-needle aspiration cytology (FNAC), management modality like the type of antithyroid and type of beta-blocker used, electrocardiography results, and echocardiography findings.

### Biochemical assessment of thyroid hormones

Our laboratory uses a VIDAS® thyroid panel with a single dose, ready-to-use reagents to perform thyroid-stimulating hormone (TSH), free T4, and free T3 assays. Blood is taken using a Gold tube. It will be allowed to clot for 30 min in a vertical position and will be centrifuged within 2 h. Then, the extracted serum will be analyzed based on the Enzyme-Linked Fluorescent Assay technique. However, our laboratory does not do Anti-TPO and Anti-Tg assays that are used to diagnose autoimmune disorders of the thyroid gland like Graves’ disease. As a result, the diagnosis of Graves’ disease was made clinically without an antibody test. Graves’ disease was considered If a patient has symptoms and signs of thyrotoxicosis, diffusely enlarged thyroid gland which has rubbery consistency without nodularity, and FNAC result that supports Graves’s disease. Our laboratory’s normal range of thyroid function tests is as follows.

- TSH: 0.5-5 Pmol/L.
- FT4: 10.6–19.4 Pmol/L.
- FT3: 4-8.3 Pmo/L

Therefore, thyrotoxicosis will be diagnosed if a patient has symptoms of thyrotoxicosis and her/his.

TSH is < 0.05 Pmol/L, FT4 is > 19.4 Pmol/L, and/or FT3 > 8.3 Pmol/L.

### Data analysis

Data entry and analysis were performed using Statistical Package for Social Science (SPSS) version 23. Categorical variables were described using frequencies and percentages. Continuous variables were also described using an appropriate combination of measure of central tendency and measure of dispersion. Odds ratio with its 95 % confidence interval and p-value were calculated by running logistic regression to identify factors associated with dilated cardiomyopathy. Variables with a p-value less than 0.05 during bivariate analysis were selected for multivariable analysis. Statistical significance was declared at *p*-value < 0.05.

### Ethical consideration

 Ethical clearance was obtained from the Ethical Review Committee of Mekelle University, College of health sciences with ethical clearance number ERC: 1408/2018. After explaining the purpose of the study, informed consent was obtained from the participants. Confidentiality of information was also assured by removing medical record numbers and replacing them with codes.

## Results

A total of 200 patients with thyrotoxicosis who fulfilled the inclusion criteria were included in this study. Those accounted for 82.3 % (200/243) of patients with a thyroid disorder who visited the endocrine clinic.

### Socio-demographic characteristics and disease category

There were 178 (89 %) females with a male to female ratio of 1:8.1. The age of patients ranged from 18 to 77 years. The mean age at presentation was 45.2 ± 14.3 years. More than half (58.5 %) of patients were unable to read and write. Toxic multinodular goiter accounted for 51.5 % of the cases (Table 1).

**Table 1:** Socio-demographic characteristics of patients with thyrotoxicosis at the endocrine clinic, ACSH from December, 2017 to June, 2018

Variables		Frequency (*N* = 200)	Percentage (95 % CI)
**Sex**			
Female		178	**89.0** (83.8–93.0)
Male		22	**11.0** (7.0-16.2)
**Age (in years)**			
18–40		83	**41.5** (34.6–48.7)
41–60		80	**40.0** (33.1–47.1)
> 60		37	**18.5** (13.4–24.6)
**Residence**			
Urban		100	**50** (42.9–57.1)
Rural		100	**50** (42.9–57.1)
**Educational status**			
Higher education		30	**15.0** (10.4–20.7)
Secondary school		25	**12.5** (8.3–17.9)
Primary school		18	**9.0** (5.4–13.9)
Able to read and write		10	**5**.0 (2.4-9.0)
Unable to read and write		117	**58.5** (51.3–65.4)
**Income**			
< 1000 ETB (< 20 USD)		106	**53.0** (45.8–60.1)
1000–3000 ETB (20–60 USD)		46	**23.0** (17.4–29.5)
> 3000 ETB (> 60 USD)		48	**24.0** (18.3–30.5)
**Primary diagnosis**			
Toxic multinodular goiter		102	**51.5** (44.3–58.6)
Toxic nodular goiter		75	**37.5** (30.8–44.6)
Graves’ disease		18	**9.0** (5.4–13.9)
Other		5	**2.5** (0.8–5.7)

*ETB *Ethiopian birr, *USD *United States Dollar

### Fine needle aspiration cytology

A total of 129 (64.5 %) patients were subjected to fine-needle aspiration cytology (FNAC). The most common FNAC finding was colloid Goiter 104 (80.6 %) followed by atypia of undetermined significance (AUS) 8 (6.2 %) and autoimmune thyroid disease like Graves’ disease 13 (10.1 %) (Table 2).

**Table 2:** FNAC results of patients with thyrotoxicosis at the endocrine clinic, ACSH from December, 2017 to June, 2018, *N* = 129

Cytology	Frequency	Percentage (95 % CI)
Colloid goiter	104	80.6 (72.7–87.0)
Autoimmune thyroid disease (AITD)	13	10.1 (5.5–16.6)
Atypia of undetermined significance	3	2.3 (0.5–6.6)
Other^a^	9	7.0 (3.2–12.8)

^a^other includes adenomatoid goiter, lymphocytic thyroiditis, granulocytic thyroiditis, thyroid cyst, nodular goiter, follicular lesions of undetermined significance, follicular neoplasm, and papillary carcinoma

### Clinical presentation of patients with thyrotoxicosis

The most common symptom was palpitation which was present in 171 (85.5 %) of patients followed by fatigue 138 (69.0 %) and heat intolerance 118 (59.0 %) while the most common sign was anterior neck swelling 186 (93.0 %) followed by tachycardia 86 (43.0 %), exophthalmos 35 (17.5 %), and tremor 35 (17.5 %) (Table 3).

**Table 3:** Symptoms, signs, and cardiac investigation findings of patients with thyrotoxicosis at the endocrine clinic, ACSH from December, 2017 to June, 2018, *N* = 200

Clinical features		Frequency	Percentage (95 % CI)
Symptoms			
Palpitation		171	**85.5** (79.8–90.1)
Fatigue		138	**69.0** (62.1–75.3)
Heat intolerance		118	**59.0** (51.8–65.9)
Irritability		110	**55.0** (47.8–62.0)
Hyperactivity		104	**52.0** (44.8–59.1)
Sweating		89	**44.5** (37.5–51.7)
Weight loss		70	**35.0** (28.4–42.0)
Increased appetite		66	**33.0** (26.5–40.0)
Difficulty of swallowing		10	**5.0** (2.4-9.0)
Dyspnea		10	**5.0** (2.4-9.0)
Abortion		6	**3.0** (1.1–6.4)
Dysmenorrhea		4	**2.0** (0.5-5.0)
Signs			
Anterior neck swelling		186	**93.0** (88.5–96.1)
Tachycardia		86	**43.0** (36.0-50.2)
Exophthalmos		35	**17.5** (12.5–23.5)
Tremor		35	**17.5** (12.5–23.5)
Lid lag		2	**1.0** (0.1–3.6)
Lid retraction		1	**0.5** (0.0-2.8)
Echo (*n* = 89)			
Dilated cardiomyopathy		35	**39.3** (29.1–50.3)
Normal		20	**22.5** (14.3–32.6)
Pulmonary hypertension		17	**19.1** (11.5–28.8)
Degenerative valvular heart disease		9	**10.1** (4.7–18.3)
Rheumatic valvular heart disease		6	**6.7** (2.5–14.1)
Hypertensive heart disease		2	**2.2** (0.0-7.9)
ECG (*n* = 149)			
Sinus tachycardia		71	**47.7** (39.4–56.0)
Atrial fibrillation		43	**28.9** (21.7–36.8)
Normal		35	**23.5** (16.9–31.1)
Sinus bradycardia		1	**0.7** (0.0-3.7)

Note: *Echo *Echocardiography, *ECG *Electrocardiography

The mean duration of the anterior neck swelling was 13.4 ± 11.1 years. A total of 12 (6.0 %) patients had thyroid storm. The most common precipitating factor was infection (41.7 %) followed by drug discontinuation (8.3 %) and diabetic ketoacidosis (DKA) (8.3 %). The rest had no identified precipitating factor.

Echocardiography was done for 89 (44.5 %) patients. Dilated cardiomyopathy (39.3 %), normal (22.5 %), Pulmonary hypertension (19.1 %), and degenerative valvular heart disease (10.1 %) were the most common findings. In addition, the most common arrhythmiaS detected on electrocardiography (ECG) were sinus tachycardia (47.7 %) and atrial fibrillation (28.9 %) (Table 3).

### Factors associated with dilated cardiomyopathy

A binary logistic regression was conducted to identify significant predictors of dilated cardiomyopathy in patients diagnosed to have thyrotoxicosis. After adjusting for confounding effect (i.e., during multivariable analysis), only atrial fibrillation and tachycardia were found to have a significant association with dilated cardiomyopathy. Accordingly, the odds of dilated cardiomyopathy were higher in patients who had atrial fibrillation (*AOR* = 15.95, *95 % CI*:5.89–38.16, *p* = 0.001) and tachycardia (*AOR* = 2.73, *95 % CI*:1.04–7.15, *p* = 0.040) than their respective counterparts (Table 4).

**Table 4:** Bivariate and multivariable Analysis to identify determinants of dilated cardiomyopathy in patients with thyrotoxicosis at the endocrine clinic, ACSH from December, 2017 to June, 2018

Variables /Categories	COR (95 % CI)	*P* value	AOR (95 % CI)	*P* value
Age (in years)				
≤ 40	1		1	
> 40	**2.46** (1.16–5.24)	0.019	**0.72** (0.27–1.95)	0.523
Level of free T4 (Pmol/L)				
≤ 60	1		1	
> 60	**2.99** (1.39–6.41)	0.005	**0.67** (0.25–1.80)	0.436
Atrial fibrillation				
Yes	**20.42** (8.45–49.31)	0.001	**15.95** (5.89–38.16)	0.001
No	1		1	
Tachycardia				
Yes	**5.48** (2.49–12.01)	0.001	**2.73** (1.04–7.15)	0.040
No	1		1	

*AOR*Adjusted Odds Ratio, *COR * Crude Odds Ratio

### Management of patients with thyrotoxicosis

All patients with thyrotoxicosis received PTU as an antithyroid drug and 13.0 % of them experienced its side effects (like nausea, stomach upset, headache, and so on). The mean dose of PTU given was 90 ± 20 mg, three times a day. Usually, patients are started on a 100 mg dose of PTU at a frequency of three times a day. Propranolol was the most commonly (78.5 % of the cases) used beta-blocker followed by atenolol (15.0 %) and metoprolol (2.5 %). The rest did not take any beta-blocker due to normal Free T4 level and/or bradycardia. Six (3 %) of the patients underwent surgery. Because of the unavailability of radioiodine therapy, none of the participants received radioiodine treatment. Health information was offered to 48 % of patients. Regarding the TSH follow-up interval, 56 % of patients had a follow-up interval greater than 3 months. After treatment, 51.5 % of the patients had their symptoms resolved within 3 months.

## Discussion

This study found nearly all patients with thyrotoxicosis are females and in the age of mid-forties. The most predominant cause of thyrotoxicosis was a multinodular toxic goiter. The most common symptoms and most common sign were palpitation and palpable thyroid, respectively. The most common arrhythmias detected on electrocardiography was sinus tachycardia and atrial fibrillation. Among those who had an echocardiographic examination, 40 % of them were diagnosed to have dilated cardiomyopathy. Regarding management, all patients received PTU as an anti-thyroid drug and almost all (96 %) of them also took beta-blockers.

In this study, the majority (89 %) of patients were females. The mean age at presentation was 45 years and those who were above 40 years accounted for 58.5 % of the cases. This is similar to a study done in Addis Ababa, in which 90 % of patients were females and patients above 40 years of age accounted for 59 % of the cases with a mean age of 43.1 years [1]. Another study in Ghana also showed similar results where the mean age was 41.5 and most of the patients (87.8 %) were females [19].

In this study, the leading cause of thyrotoxicosis was a multinodular toxic goiter. This finding was similar to a study done in the North-Western part of Ethiopia, which reported 54.9 % of thyrotoxicosis cases due to toxic multinodular goiter [20]. This is explained by the presence of unresolved iodine deficiency in Ethiopia with a prevalence of goiter that ranges from 35 % up to 70 % [21–24]. According to a recent study done in Ethiopia, more than half (57 %) of school children were found to have low urinary iodine levels [24]. This problem of iodine deficiency has persisted in this country despite the availability of iodized salt [23].

The most common symptom of patients with thyrotoxicosis was palpitation which was present in 85.5 % of the patients followed by fatigue (69.0 %) and heat intolerance (59.0 %). Similarly, Addis Ababa’s study revealed palpitation and heat intolerance as the most common symptoms of patients with thyrotoxicosis [1]. As well, a study done in South-Western Nigeria found palpitation and fatigue as the most common symptoms which occurred in 85.0 and 63.4 % of patients, respectively [25]. A study done in France on 1240 patients also supported the above findings and reported palpitation followed by weakness as the most common clinical presentation of thyrotoxicosis [5].

The most common sign in our study was palpable thyroid (93.0 %) followed by tachycardia (43.0 %), exophthalmos (17.5 %), and tremor (17.5 %). According to Addis Ababa’s study, palpable thyroid (goiter) was also the commonest sign of thyrotoxicosis which was detected in 99 % of the cases. Congruently, the most common sign in Nigerian study was palpable thyroid and found in 97 % of the cases [25]. On contrary, a study done in Maharashtra, India found tremor as the most common sign of thyrotoxicosis, in about 63.0 % of patients [26].

The most common arrhythmia detected on electrocardiography (ECG) was sinus tachycardia followed by atrial fibrillation and this finding goes parallel with several case studies on thyrotoxic cardiomyopathy [27–29]. This prevalence of atrial fibrillation on ECG of patients with thyrotoxicosis is also comparable to a study done in India where it was 28 % [30]. The prevalence of dilated cardiomyopathy in our study (which is around 40 %) is higher compared to other studies where it is reported to be 1 % [31]. Even taking the whole participants as a denominator, still, the prevalence is higher which is about 17.5 %. There are two main reasons for this higher prevalence. The first reason is the late presentation with a mean duration of goiter (i.e., anterior neck swelling) of about 13 years. This explanation is supported by a study done in England that found older age, male sex, and longstanding thyrotoxicosis as factors that increase the risk of dilated cardiomyopathy [31]. The second reason is that echocardiography was done only for patients with signs and symptoms of thyrocardiac diseases. In this study, the factors significantly associated with dilated cardiomyopathy were atrial fibrillation and tachycardia. Thyroid storm was present in 6 % of this study participants. This figure is comparable to a national study done in Japan where it was 5.4 % [11].

In our study, PTU was the only thionamide used to treat thyrotoxicosis. This is similar to the study done in Addis Ababa, Ethiopia where almost all of the patients received PTU [1]. This finding contradicts a survey done by members of the Endocrine Society (ES), American Thyroid Association (ATA), and American Association of Clinical Endocrinologists (AACE 6) where the most commonly used drug was methimazole (83.5 % of the cases) while the use of PTU was limited to 2.7 % [32]. This change in trend towards methimazole use in the developed world is because methimazole is associated with a high rate of free T4 normalization and fewer side effects [16, 33]. But in developing countries like Ethiopia, methimazole is hardly available and almost all thyrotoxicosis cases are treated with PTU [20]. Currently, the recommendation is to use methimazole as the first line anti-thyroid with an exception during pregnancy in which PTU is preferred because of rare reports of birth defects associated with methimazole [34]. Additionally, in life-threatening conditions like thyroid storms, PTU is also preferred since it inhibits the conversion of T4 to T3 [35]. However, PTU is not a good choice of drug in controlling severe forms of the disease because it has a poor adherence rate, and several adverse effects [36].

The interval of TSH follow up was > 3 months in 112(56 %) of the patients and it was against the recommendations by the American Association of Clinical Endocrinologists and the American Thyroid Association which recommend TSH determination every 4 to 6 weeks for all patients till normalization of TSH and T4 [32, 37]. The most common reasons not to do it based on the recommendation were limited availability of the investigation and financial constraints (more than half of the patients had a lower monthly income which is below 20 USD).

### Strengths of the study

It is the first study to assess the pattern, clinical presentation, and management of thyrotoxicosis in Tigray and can serve as a baseline for future studies related to the current topic. It has also tried to assess many aspects of thyrotoxicosis by collecting primary data using a pre-tested and comprehensive questionnaire.

### Limitations of the study

Echocardiography, electrocardiography, and cytology were not done for all patients. As a result, this study may not show the true prevalence of cytologic types and cardiac complications in patients with thyrotoxicosis. Due to infrequent TSH follow-up, this study did not address time to normalization of free T4 and TSH.

## Conclusions

This study has shown that most of the patients with thyrotoxicosis were females and more than half of the thyrotoxicosis cases happened in patients with multinodular goiter. The most common symptom and most common sign of thyrotoxicosis were palpitation and goiter, respectively. All patients took PTU as an anti-thyroid drug. Dilated cardiomyopathy was the commonest abnormal echocardiography finding in patients with thyrotoxicosis and it was significantly associated with atrial fibrillation and tachycardia. The prevalence of thyrocardiac disease was higher even taking all participants as denominator which may be attributed to the longstanding thyrotoxicosis. Overall, the findings of this study complement and strengthen studies done in another part of Ethiopia and other African countries.

## Recommendations

Patients should be given adequate health information about thyrotoxicosis and anti-thyroid drugs including their side effects. Hospitals and other concerned bodies should also avail of TSH tests and methimazole at an affordable cost. In addition, community awareness about iodized salt and iodine-rich foods should be enhanced.

## Data Availability

The datasets used and/or analysed during this study are available from the corresponding author on reasonable request.

## Abbreviations

ACSH: Ayder Comprehensive Specialized Hospital
AUS: Atypia of undetermined significance
DKA: Diabetic Ketoacidosis
ECG: Electrocardiography
ETB: Ethiopian Birr
FNAC: Fine Needle Aspiration Cytology
PTU: Propylthiouracil
SPSS: Statistical Package for Social Sciences
SSA: sub-Saharan Africa
T4: Thyroxine
TSH: Thyroid Stimulating Hormone
USD: United States Dollar
